# Site-Specific Bioinspired Architecture—A Case Study of the Allen–Lambe House by Frank Lloyd Wright: The Pragmatic versus the Naturalistic, Intent versus Realization

**DOI:** 10.3390/biomimetics8020178

**Published:** 2023-04-24

**Authors:** Richard Hyde

**Affiliations:** School of Architecure, Design and Planning, The University of Sydney, Darlington, NSW 2008, Australia; richard.hyde85@gmail.com

**Keywords:** bioinspired architecture, biomimetics, eco-mimesis

## Abstract

Research into biomimetics has focused on science and technology, often developing the innovative parts of buildings from nature, leading to a new field of bioinspired architecture. The work of Frank Lloyd Wright is presented as an early example of bioinspired architecture and explores how buildings might be more broadly integrated into their site and environment. Integrating architecture, biomimetics and eco-mimesis as a framework to look at the work of Frank Lloyd Wright, provides a new understanding of his architecture and directions for future research into the ecological design of buildings and cities.

## 1. Introduction

A recent urban manifesto points to the continuing increase in the pressure of human development on the environment which has now been a concern for many years; the pressure is now increasing. The environment is caught up in a range of pragmatic responses, economic needs and the maintenance of the human quality of life [1]. This is re-energizing thoughts about how the design of the built environment might help shape the response to this pressure [2]. Hay argues that environmental thought shaped around the state of the environment provides a new ecological paradigm that seeks, above all, to develop a higher value for nature and to prioritize conservation over the utilization of natural systems [3]. In a historical analysis, he posits that a romantic view of nature developed as a reaction to the Industrial Revolution and its social and physical disruption. Hence, this view of nature is displaced by new activism in support of that nature and humanity are inextricably linked and that this notion demands new scientific and ecological insights. More recently, social beliefs of the low value of nature, the importance of economic growth value over environmental protection, and the lack of limits to growth are three barriers to wide social acceptance that provide the remaining context [3].

For design disciplines, these complications challenge the integration of the ecological paradigm into architecture. Where this has occurred, it is through the process of ecological design. Research by Lydia Kallipoliti reports on a historical analysis of the evolving concepts and perspectives of naturalism, which form the basis for ecological design. Three periods are identified [4]:

1. The first period was described as ‘searching for roots’ and started with the term ‘ecology’ by Ernst Haeckel in 1866. He started with ‘oekologie’, meaning ‘home’ in Greek; hence ecology became known as the study of the relationships between living organisms, the biotic and abiotic environments that they inhabit. Much of this work involves the categorising of natural elements. Early American philosophers and writers took a transcendental view of this process. This creates a perception of nature as part of the American national identity and draws on divine spirituality through the inspiring organic quality of nature. This line of argument drew attention to ongoing human-induced environmental destruction. Architects such as Wright are seen as immersionists, drawing on this vision for their architecture.
2. The second period, ‘synthetic naturalism’, emerged toward the end of World War II and continued until the end of the 20th century. This viewpoint saw ecological design as a system approach to allow the equitable use of natural resources for industrial processes. Ecological design is seen as a tool for this purpose. In 1960, the reframing of ecological design as synthetic naturalism emerged; rather than using natural systems, these were now emulated. For example, climatologists such as Olygay advocated using natural systems to heat and cool buildings; this approach was displaced by machines and air conditioning which arguably created a synthetic environment.
3. The third period, called ‘dark naturalism’, has been evolving in the first part of the 21st century. However, the extent to which the systems approach—natural or synthetic—has been successfully implemented is debatable. This has heralded a belief that human needs take precedence over the existence of nature. Hence, Kallipoliti development of the concept of dark naturalism seeks to avoid this dilemma and led to the development of eco-perspectives to address deteriorating planet-scale environmental conditions such as climate change. Contextualist perspectives in architecture may be of assistance here, such as critical regionalism. This perspective tempers the pressures of global impacts on the resources of a particular region to create a link to ecology and sustainable architecture.

Further new naturalistic perspectives have emerged that mimic nature, such as bioinspired architecture and biomimicry. A review of the research by Uchiyama Yuta, Eduardo Blanco, and Ryo Kohsak on advancing research in biomimetics is seeking design innovative strategies and solutions to address architectural and urban design issues at varied scales from the molecular level through to architecture and to the urban scale. They conclude that biometric methods can operate at these broader scales using both traditional and scientific knowledge, but that the social implementation of biomimetics is limited [5].

Pedersen Zari reports that the social acceptability of biomimicry lies in its potential affordance of sustainable and regenerative architecture through the emulation of nature in design; however, design methods need further development. She argues that biomimetics organizes the natural world, ecologically, as several levels—organisms, behavior, and ecosystems; as part of its investigative process, it is open to the use of architecture for integration into its design process [6]. 

One of the opportunities for this direction is the concept of ‘bioinspiration’ based on biomimetics. Questions emerge as to what it constitutes and its potential for integrating architectural and natural systems. A recent historical analysis of bioinspired architecture by Ripley and Bushana examines Frank Lloyd Wright, renowned for his work on organic architecture, which centers on the harmony between man and nature, a new field called bio-architecture based on biomimicry. Two definitions are provided:

- Organic architecture emphasizes the site-specific nature of the architecture and incorporates architecture into nature, essentially at the broader scale of nature as a whole.
- Bio-architecture emphasizes the concept of architecture as a whole and the incorporation of nature through mimicry, but only at the scale of the whole building and its site [7].

This observation suggests that the objectives and scope of bio-architecture are very different to that of organic architecture, despite using the same source of inspiration:

- Bioinspiration biomimicry can create a bio-architecture integrating parts of nature into architecture.
- Bioinspiration through organic architecture integrates architecture into nature using the site and its broader environment.

**Figure 1:**
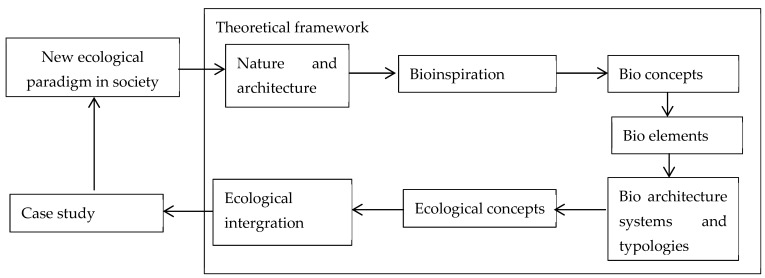
Conceptual diagram of the study issues.

The study issues are shown in Figure 1. The first part of the paper explores the constituents of bio-architecture—its bioinspiration, bio-concepts, and ecology—and its relation to architecture—its elements, systems, typologies, and integration. The second part uses this theoretical framework to examine a case study of organic architecture, emphasizing the biomimetric perspective. The final part compares the two approaches and their implications for the design of cities.

The scope of this study focuses on design thinking ranging from the concepts of nature to their realization in the built form. This involves exploring the design intent and alignment of the site’s bio- and ecological systems and the city’s broader environment, which is currently shaped by pragmatic forces.

The study’s limitations are that it uses a case study methodology combined with a descriptive approach, which sacrifices breadth for depth. This methodology often leads to more questions being posited than it necessarily answers. However, these form the basis for further research and design reflection on strategies for advancing the new ecological paradigm in society.

## 2. Theoretical Framework

### 2.1. Bioinspiration, Biomimicry, and Architecture

Exploring the notion of bioinspiration definitions shows a plethora of opportunities for biomimetics:

- Bioinspiration—using phenomena in biology to stimulate research in non-biological science and technology—is a strategy that suggests new areas for research [8].
- Bioinspiration involves taking principles from biological systems and applying them to technological and design problems. As global challenges become more complex, we increasingly draw inspiration from biological systems to find new solutions [9].
- Biological systems in nature have inspired the study and design of engineering systems and modern technology [10].

Exploring the integration of parts of nature into architecture through biometrics creates some challenges and opportunities. Research by Imani and Vale points out that because biology and architecture are different domains, it is difficult to transform biological knowledge into design principles and concepts [11]. They aim to provide a thermo-bio-architectural framework to provide an in-depth understanding of thermoregulatory strategies in animals and plants in order to link these to equivalent solutions in architectural design. 

Whilst this research is about facilitating the means of biomimetic design, a way of understanding the ends to those means is also needed. The authors point out that whilst biomimicry may provide innovation, this may not lead to a more sustainable future [12]. 

### 2.2. Bio-Concepts and Nature

Bioinspired design is an umbrella term for many of the bio-concepts related to architecture and engineering; it is a proxy variable for investigating the phenomena of nature:

- ‘biomorphic’—looks like nature;
- ‘bio-utilization’—uses nature;
- ‘biomimicry’—functions like nature [13]; ‘biophilia’—affiliations with nature;
- ‘bioclimatic’—responds to nature.

Additional bio-concepts, such as biophilia and bioclimatic approaches, should also be mentioned. These relate to how humans experience nature regarding biotic and abiotic constituents. These are founded on the innate human experience of nature at a deep physiological and psychological level. Wilson, in his book, argues that biophilia is a hypothesis that proposes that humans have a connection with nature. Part of being human is the filiation with other organisms and nature; in the same way, our buildings are less human without other organisms [14]. This observation is the basis of an environmental conservation ethic that underpins bio-concepts and nature.

However, Hoyos et al more recently argues bio-affiliations emphasize the importance of nature as a beneficial part of the human condition, and there is growing scientific evidence of the positive effects of nature on health. [15].

Ryan et al. defined biophilic design patterns as those which provide architectural guidelines for use in design to aid the affiliation of the occupants with nature [16]. Affiliations are mainly derived through the sensory perception of natural systems and spaces; however, architecture can be derived from nature by abstraction. 

Space patterns created in nature are also a source of biophilic ’bonding’ between humans and nature. They emphasize the importance of spatial patterns afforded by nature and their connection with beneficial experiences as an outcome of Biophilic design such as…

- Nature in the space patterns- visual and non-visual connection, variability in sensory stimuli, light, thermal and airflow, presence of water and natural systems
- Natural analogues patterns- biomorphic forms, material connectivity and contrasts between complexity and order of systems
- Nature of the space patterns- prospect view of nature from a point of refuge, sense of mystery, risk and peril [17].

The bioclimatic approach, pioneered by Olgyay, perhaps more established within the architectural discipline, provides a methodology for design considering the climate to create regional architecture. By increasing the goodness of fit between the site and the climate, adaptations can be made to a building in order for it to operate with natural systems for better human comfort [18]. This responsive process is used for the passive design of buildings.

Bio-utilization, like the bioclimatic approach, is the connection between a building and its site. Hence, the utilization of living organisms on the site is a central part of its environmental design since bio-utilization describes the act of using living organisms with human systems to achieve a range of functions, such as the climate control of buildings. Pedersen Zari, notes bio-assistance functionality creates interdependence between the abiotic and biotic systems [19].

One of the foremost challenges and opportunities is to interconnect these ‘relatives’ of biomimetics through the architectural process for the development of building elements.

### 2.3. Bio-Elements and Ideation

However, there is an ongoing discussion in the biomimetic research literature about how bio-concepts are to be used in architecture to design elements. This is framed around which biological constituents are used and the design methodologies used to transform them. For example, a review by Yuta Uchiyama, Eduardo Blanco and Ryo Kohsak notes that research trends show the use of biometric design for elements such as materials, structures, and systems such as façades and biophilia [5].

Research studies have started by developing frameworks to investigate parts of natural phenomena integrated into architecture and engineering. Badarnah and Kadri suggest that there are two approaches to ideation:

1. The top-down approach, in which biomimetics uses an analogy to derive solutions from nature resolve design issues;
2. The bottom-up approach, in which biomimetics uses induction from design issues to derive solutions from nature [20].

One observation is the highly complicated utilization of what could arguably be called a generic idea pathway of the biomimetic design. Research by Romain Farel and Bernard Yannou into teaching bioinspired ideation to engineering students found that part of the challenge in biomimetics is finding compatible sources which can be usefully transferred between the two domains of biology and design, and another challenge is how they form inspiration as part of this transformation process [21].

The lessons learned from this study suggest that this approach may be overly directional; working from the solutions in the ‘biological’ space to solve problems in the engineering space and vice versa may confound creativity. However, the authors started using an iterative process considering that these two spaces synchronously might be more productive. Outcomes point to designers needing more help with the problem-based approach over the solution-based approach, and how the inspirational ideas developed in the project are used for product realization. Using this direction may build on Farel’s work and provide insight into harmonizing biometric and architectural systems ideation in a practical sense.

If biomimetics is placed in a broader ecological design approach, a contextual perspective of nature can be entertained, as previously observed by Lydia Kall Kalli. This means designers look specifically at the site and its location in a regional context as the solution space for biomimetic projects.

### 2.4. Building Typologies and Bio-Architecture Systems

In recent years, building typologies have emerged which have undermined the contextual approach. Cullen, in his introduction to Randal Thomas’s book *Environmental Design*, noted that the making of buildings has changed to a highly serviced typology, that is, sealed, air-conditioned, compact forms which are operated using fossil fuel energy. He argued that once the building is sealed, the design must accommodate a host of considerations that fundamentally change the basis for the form and aesthetics of the architecture. Cullen advocated for an environmental approach as a basis for architecture, shaping buildings according to the environmental conditions in and around buildings, leading to an environmental typology as a basis for architecture [22]. Hence, a debate in architecture exists about how the relevant typologies in architecture should be shaped by the environment or shape the environment through associated impacts.

Yeang argues that the availability of relatively inexpensive energy sources, such as fossil fuels, has significantly impacted the quality of life of humans globally and the actual making of architecture. However, fossil fuel energy is no longer a preferred option as a basis for architectural typologies; a new typology that is bioinspired and ecologically driven is needed [23]. This direction points to a link in the literature in the biosystems view of architecture, including ecosystem behavior, flexibility, and adaptability as attributes of the sustainability of buildings [24].

Duffy set the groundwork for this approach arguing that the functional fit of buildings requires the integration of the shell (building envelope), the scenery (the internal subdivisions), and the set (the furniture) [25]. Leeman, Adrian, and Bill Bordass, advanced this further conceptualizing that physical systems in buildings can be viewed as a series of layers organized hierarchically. The interdependent nature of a building’s functional program requires different configurations of these systems, hence allowing for the adaptability required in the examination of future needs to avoid unintended consequences. The concept of adaptable multifunctioning space became necessary 

This natural hierarchy started with four main constructs comprising the site, shell, and scenery and was extended to several constraints, dependencies, and interactions, with resulting adaptions between each other and the whole:

- site—geographical setting, location, environmental services;
- strategy—concept, building massing and form;
- shell—building envelope;
- structure—foundations and bearing structure;
- skin—external surfaces;
- services—HVAC, electrical, mechanical, and hydronic systems;
- space plan—a spatial organization;
- scenery-layout of internal partitions;
- set—furniture and equipment;
- stuff—occupants’ belongings.

They note that physical systems at the top often form constraints and opportunities for those at the bottom and that all of these have different life cycles. This approach sets up a design approach for discussing economic, social, and environmental issues between occupants and businesses [26]. 

Research by Estaje reports an unintended consequence of the systems approach is ecological disruption [27]. He suggests that this framework can be extended into a wider scale of dependencies and interactions based on the work of Harbraken and Brand [28,29]. The aim is to describe physical systems that are flexible and adaptable over time, thinking through the dependencies and interactions in wide spatial scales—city, neighborhood, site, shell, scenery and set—in the design and operation of buildings [25]. Design research by Li et al. has utilized this approach to include cultural, territorial, and natural systems in the model to provide sustainable building regeneration [30]. 

### 2.5. Ecological Concepts

One observation is that the concept of the ‘ecosystem’ in ecology is like the concept of the ‘site’ in architecture. The term ‘site-specific’ architecture relates to the proposition of using the ecosystem concept as a source of bio-inspiration for architecture. Three levels of biomimicry were defined by Pederson Zari for architectural design, comprising three levels:

- organism level;
- behavioral level;
- ecosystem level.

In a review of biomimetic design approaches, She argues that the ecosystem approach offers several advantages. Sometimes called eco-mimicry, it is relevant when the objective is the well-being of ecosystems and people as means of deriving a sustainable form that creates a pathway to more resilient buildings. It also incorporates addition levels such as the organism and behavior levels which include sustainable building methods and bio-assisted systems, applicable at a range of temporal and spatial scales, leading to improved environmental performance. However, implicit in this approach is an in-depth understanding of the ecological processes and an interdisciplinary team challenging the conventional approach to design. This approach may offer a more holistic way of examining bioinspired buildings; hence the methodology for this project evolved from this framework. Through investigation at the organism level, it is possible to explore the wider design implications by integrating additional perspectives. Conceptually, the ecosystem in which the building is situated comprises several organisms which have collective behaviors both amongst themselves and with the environment.

She proposes an ecosystem approach. She poses the question of what may be included in architecture if it is to be called ‘biometric’: ‘The design may be… biometric for example in terms of what it looks like (form), what it is made from (material), how it is made (construction), how it works (process) or what it can do (function) [6]. This framework is adapted in the next section as seen in Table 1.

### 2.6. Ecological Integration

One observation is that the framework draws on fundamental unifying principles of ecology which can aid the aims of mimicking ecosystems and integrating sites and buildings with the ecosystem [31]. This involves exploring how these principles are used in the design, the extent to which they lead to strategies that serve as a means and to what end in the problem-solving process. Hence form and function may be thought of as ends whilst material, construction and process are means. This is important as in building design, additional ends such as human functions require dovetailing with the ecological. 

The question is how to conceptualize ecosystems as models for social systems and for design. The study of Margalef uses a parametric approach to derive the unifying principles in ecology:

- ecosystems have a structure of parts or elements that together make a whole;
- the interrelations between the constituent elements are the basis of the structure, and these form the model’s parameters;
- measuring an ecosystem by the degree of complexity of these parameters is possible;
- hence, more mature ecosystems have more parameters and, therefore, more complexity.

Magalef’s study reports on the essential changes in the behavior of ecosystems according to the principle of succession, i.e., the shift from less to more mature systems using energy and biomass.

The first principle observed is that the rate of an ecosystem’s increase in complexity and maturity is coupled with a growth in biomass but a reduction in energy flow. The second principle is that there is always a flow of energy from the less mature to the more mature systems. Furthermore, in mature ecosystems, biomass and energy use stabilize, and the parts of the ecosystem develop synergistic functional relations, i.e., they mutually benefit each other.

The study shows that large structures such as cities can be modelled this way, with rural areas seen as less mature than urban centers with greater complexity; however, like their ecological counterparts, they are also susceptible to small changes in environmental conditions which degrade their stability. Therefore, these principles are integrated into examining the case study in the present work [31].

Cuce et al. comment that while biomimicry may be directed to the creation of novel forms of architecture, there are questions about integrating these forms into natural systems and their sustainability. These observations point to the need to align architectural intent with ecological purpose. Hence, they recommend that biomimetics be investigated using a more holistic methodology, including the discussion of ecological integration to provide a sustainable and more regenerative architecture [32].

## 3. Case Study

**Figure 2:**
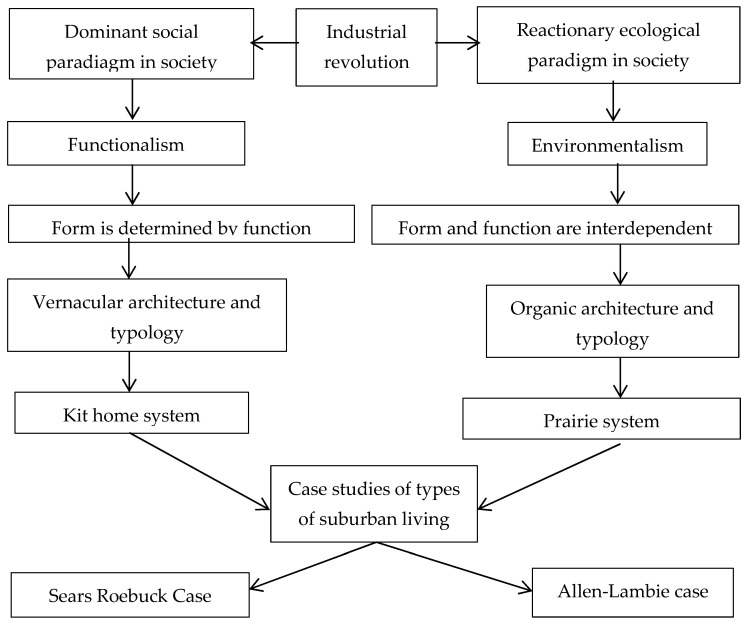
Conceptual diagram of the case study argument.

The following analysis is based on ongoing research into the work of Frank Lloyd Wright, involving visits to his notable buildings and exploration of the literature surrounding the growth of his ideas. Figure 2 shows the development of the analysis coming from the source ideas of how Wright aimed to differentiate his architecture from the prevailing discourse at the time, aligning with the reactionary environment paradigm against the social and environmental disruption of the Industrial Revolution. Using some of the selective ideas, it is possible to examine how these shaped his architecture and how this compares to the vernacular which sits more in the accepted orthodoxy.

Wright was part of a group of architects trying to create a new indigenous architecture through design with nature and to create a new vision for suburban living. Called the ‘prairie school’, the new approach was significant and enjoys ongoing investigation as part of World Heritage listings [33].

Wright was a member of this school. He developed his own typology called the ‘prairie houses’, of which the Allen–Lambe house is a case, one of the last of its type before moving new types.

On visiting the house in early 2022 as part of fieldwork investigating Wright’s legacy, it was evident that it was unusual since it was built in an urban setting. Now a museum contributing to the historical conservation of American heritage, one was able to investigate the inside and outside of the building, understanding more of the integration of Wright’s ideas [34].

The notion of the significance of integrating architecture into nature Wright has also been noted by numerous authors for its contribution to American culture [35]. One of the challenges of this study was the diverse collection of material documenting his work; perhaps the most cohesive is Wright’s own critical writings, which were collated and edited in recent years by Bruce Pfeiffer [36]. He notes that Wright’s article on ‘*The Cause of Architecture*’ in *Architectural Record*, 1908, provides a detailed explanation of his work and the prairie houses; it predates the present case study. 

This is complemented by additional disciplinary perspectives which focus on typological developments in his work and the broader environmental implications [37]. Hence, first, the ideas underpinning Wright’s work are provided; second, an examination of the prairie house type is provided, followed by a more detailed review of the Allen–Lambie house as a case that provides information regarding its type, and hence, provides an example of a realization of the prairie vision. This is also examined against the backdrop of the vernacular, which is part of the orthodoxy, placing Wright’s work in context. The prairie vision and the model for modern suburban living represent the intent, and the case represents realization.

### 3.1. Sources of Bioinspiration, Bio-Concepts, Ideation, and Interests

Examining Wright’s work historically, this is divided into four periods and different types of houses as reported by Thomson.

- early period (pre1901)—a derivative of his work with other architects, early prototypes integrating his design ideas;
- prairie period (1901 onwards)—new house type characterized by organic architecture principles and the use of natural materials and harmonizing of the building with the site;
- textile block period—cast concrete types inspired by Mayan architecture;
- the Utsonion period (1935–1955)—low-cost types built by clients which could be prefabricated [38].

#### Propositions, Function, and Linear Abstraction

Before these periods, his biography by Secrest notes that his formative years were critical to his ideas about nature when his passionate affiliations with nature were formed through his life and family. These factors should not be underestimated in the motivation of the ideation processes, He remained true to his propositions throughout his career [39]. Furthermore, in ‘*The Cause of Architecture*’, he places the ‘sense of the organic as indispensable to an architect.t’ From this, he developed six propositions for architecture:

1. Simplicity and repose—this applies to rooms, openings, details, pictures, and furniture (preferably built-in);
2. Many kinds of houses to reflect the individual;
3. A building should grow easily from the site and to harmonize with its surroundings;
4. Colors require the same conventionalizing process;
5. Bring nature into materials;
6. A house must have character.

**Figure 3:**
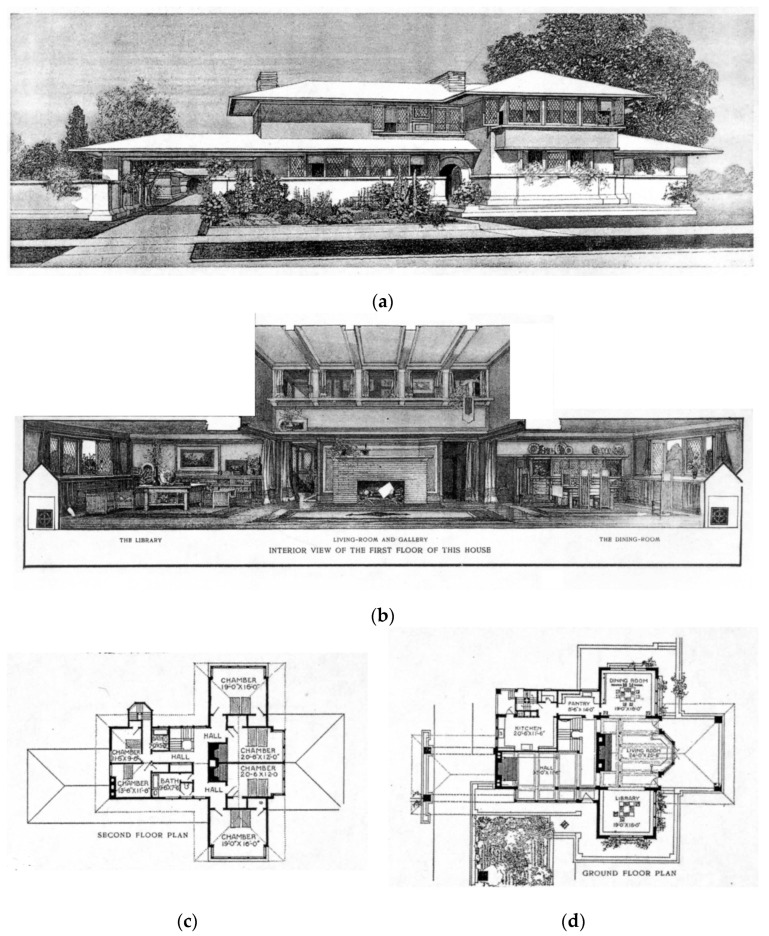
Prairie house type, as a model for suburban living contained, symbolic, landscapel and technological elements with natural backdrop; (**a**) exterior perspective; (**b**) interior perspective showing open plan and open section; (**c**) second-floor ‘T’ shaspred plan; (**d**) ground floor cruciform plan. Adapted from, Wright, Frank Lloyd, “*A Home in a Prairie Town*”, *Ladies Home Journal*, February 1901 ©. (published monthly by The Curtis Publishing Company, Philadelphia), http://www.steinerag.com/flw/Periodicals/LHJ.htm. Accessed on 10 January 2023.

These propositions are expanded in the text; they flow from the concept of the organic, but they also reflect additional interests such as new technology. Additional ideas sought to differentiate his architecture from his peers, particularly in the early period but also reinforced in the later periods. Two notable ideas are his attitude to function and line art as a form of abstraction. Wright argued against the notion of a deterministic view of function, i.e., that form follows function. He saw Functionalism as having limitations because it gave little opportunity for creativity and led to prescriptive professionalism [40]. (see Figure 3). 

Hence, he argued that the inclusion of both form and function allows a broader interpretation and paves the way for more interdependency between nature and building. Furthermore, the use of the linear abstraction of the form indicates an alternate approach to reflecting domesticity and respecting the site in his architecture, as compared to the vernacular housing of the time [41]. Through this comparison, more detail of how the ideas shaped a model for suburban living creates a building form that better integrates with the site and is serviced by the environment.

This model comprises symbolic and technological elements. Biomimetic abstraction (a, b, and c) and bio-utilization are central concepts: (a and b) at the levels of the façade, adjacent landscaping, and ecosystem (a, c, and d), the integration of symbolic elements inform the spatial organization, providing what Wright calls ‘little resistance’ to modern living. This house can be opened vertically and horizontally through adaptable screening, but can also close into a more cellular organization.

### 3.2. Prairie House Type: A Model of Suburban Living

Hence, the prairie house type was conceived from this model for suburban living; this is manifested in many of his plans and drawings of the following example of his work as repoted by Thomson [38]. It is from this evidence that an analysis of the techniques he used can be developed.

#### 3.2.1. Ornamentation, Bio-Elements, and Nature

Wright, in the early period of his career, differentiated his art through the notion of ‘ornament.’ David Hanks discusses the development of ornamentation based on nature. Drawn from design theories of the time, ornamentation should be derived from a systematic analysis of organisms:

- how the whole was dependent on the integration of the parts;
- how form reflects its progress and growth;
- the adaptations through use and the environment.

He discusses the development of an ornament, like the discussion of bioinspiration and mimetics, and the creation of bio-elements. Here, we see this as Wright positioning an architecture constrained by what nature can provide and the machines, i.e., the technology, could make. Additionally, the creation of an organic architecture required a change in the notion of the façade, not as a line of enclosure but adapted as a bio-element more akin to a filter allowing an interdependency between the inside and the outside. It is entirely possible that external conditions could be experienced inside when favorable. These bio-elements were ornamented. Comparisons between the ornamental work of Wright and his peers show this approach. The basis of this analytical approach breaks the biological element into its abstract shapes, arriving at geometry using abstraction and interpretation to inform the design [42]. 

The roof form is an ornamental example of this process, reportedly an analogy of the prairie using dominant horizontal lines which create a geometric abstraction. However, these were reinterpreted in such a way that it can shelter the internal space but not dictate their form. Goldsmith argues that interpretation is important to architectural design, whereby the adaption of several parts of the whole is used to create a unique and stable relationship in the form of the build [43]. This resembles the techniques used in the development of the prairie system.

Once the process is understood, it becomes relatively easy to recognize the underlying analogies, for example, in the windows and the other elements of the building, through ornamentation to which Wright alluded, as opposed to figuratively using natural elements, as in other architectures. Geometric abstraction allows an understanding of the composition of the whole design through its parts. 

Hence, in biomimetic terms, an observation can be made that Wright works in the solution spaces of nature and technology. The problem phase is in the process of abstraction; hence, for Wright, it is a process of finding a way for nature and technology to work together. This is now becoming an important goal in biometrics [44]. An observation can be made that a design is created by nature and with technology; however, this duality oversimplifies the challenges in addressing the needs of others, i.e., the clients and the site.

#### 3.2.2. The Bio-Architectural System

**Figure 4:**
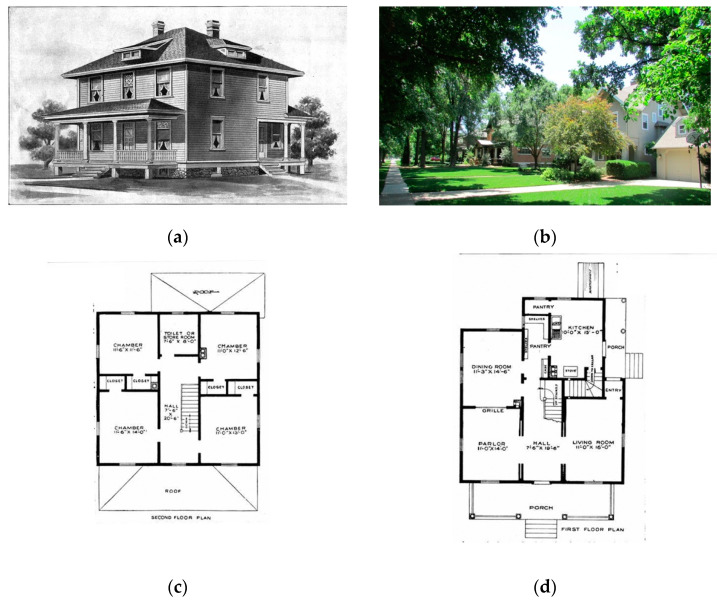
A vernacular Sears Roebuck 4 square house, system-built kit home. (**a**) site planning and built form; (**b**) second-floor plan; (**c**,**d**) ground-floor plan. Adapted from, Sears, Roebuck & Co., public domain, via Wikimedia Commons https://commons.wikimedia.org/wiki/File:SearsHome102.jpg. Accessed on 10 January 2023.

Architectural theorist Richard A. Etlin points to the prairie houses by Wright as one of the first examples of the emergence of an architectural system. Etlin’s research argues that, at the time, architectural thinking was moving towards modern architecture, based on principles, rather than being based on forms from the past, as shown in Figure 4. Such principles comprise some of the emerging interest in the landscape and the romantic idea of the ‘organic’ and engagement with the ‘poetic’ aspects of life, which are the intangible dimensions architecture might engage with. These are bound up with symbolism and the human experience of natural elements such as sunlight and water.

He points out that Wright created a bio-architectural system. This developed from the previous historical analysis that architecture arose over time from the cultural use of contemporary materials and construction; the architecture system comprises construction, form, and decoration. Wright synthesized these into two aspects:

- the symbolic elements, represented by aspects of American life: the hearth, the protective roof, a building built on a firm base, and screen walls;
- the form and decoration, derived from innovative construction.

This prioritizes the internal space, associated space planning, and decorative elements derived from abstractions from nature and the prairie ecosystem. Elin further points out that the main intention of Wright’s work is the imitation of nature, rather than its representation [45]. This aligns with bio-inspired objectives mimicking biological and ecological systems.

#### 3.2.3. Biosystems and Architectural Typologies

Wright’s architecture is a naturalist analogue of its setting; the site is adjusted to the needs of modern living. The symbolic elements are seen as constraints on the design process as an architectural system does not formally dictate how this should be accommodated. His response, some have argued, is that he provided a modular spatial organization in the architecture called a ‘grammar’. It is this that distinguishes it from contemporary designs [46]. The realization of his architectural system is discussed by Storrer through comparison with the vernacular and the prairie architecture, describing several differences between these types [47]. Figure 4 shows that the vernacular housing typology was also an architectural system of its time; having a compact, three-story form, the cellular spatial organization comprises a central hall with rooms and chimneys on the outer perimeter.

The timber kit home version; these prefabricated houses were extremely popular, functionally servicing expanding commerce across cities and being easily assembled using carpentry and farming skills [48]. Further analysis of this type (a) The overall form appears largely agnostic to the site and climate: (b) site planning addresses road service entry and backyards with differing landscaping and ecological growth; (c) a compact platform is used, i.e., a spatial organization with four main rooms per floor serviced by a central hall and stairs in a bilateral symmetry; (d) the form is extruded vertically from the plan into a box, and transition spaces between the inside and outside comprise porches that are additive, moving to asymmetrical organizations at the rear.

The prairie housing system was an alternative to the above. Storre carried out an extensive analysis of Wright’s work, including the planning of houses; the notable characteristic of the approach was the unit system. 

Geometric patterns inform spatial planning building design; the grid organizes the dimensional and aesthetic constituents of the spatial organization, and the cantilever informs the integration of the spatial planning and building envelope [47]. Hence, a bespoke solution can be created for each site and each client within the bioinspired conceptual umbrella. This allowed the spatial organization to vary with the site and program constraints while still maintaining the original intent through the organic ornamentation of the roof form, façade composition, hearth, and interior subdivision

### 3.3. Allen–Lambe Case

Holistically, all the prairie houses are the same type; however, further observation of the Allen–Lambe case and the vernacular shows they are all different. How so?

#### 3.3.1. Ornamentation Applications

The ornamental applications are similar in the prairie system and the case study, reflecting the analogies to natural systems that order the construction system. However, in the vernacular case, the expression is functional, expressing the timber construction system per se, ordered by the fabrication process.

**Figure 5:**
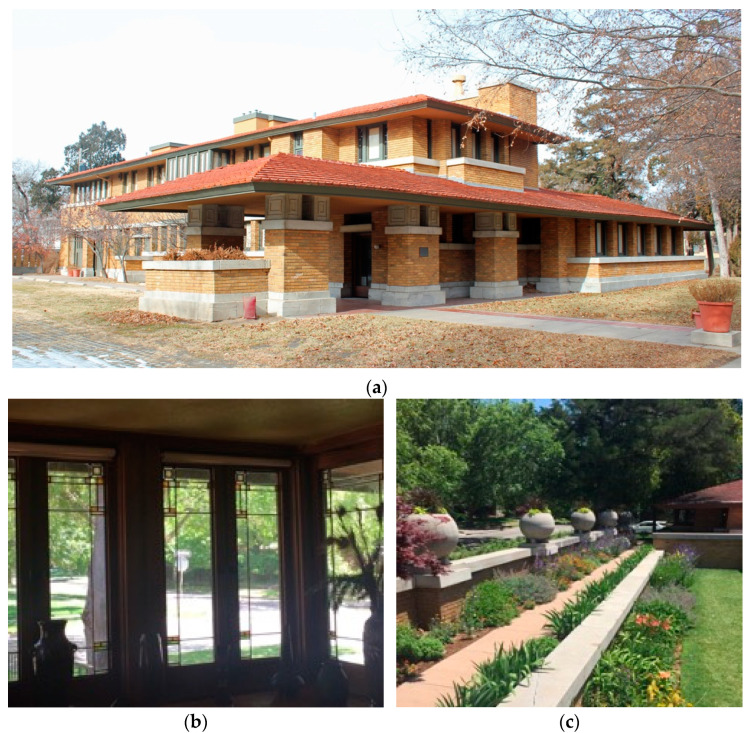
Allen–Lambe case. (**a**) Perspective view from the street ornamentation abstractions from the Prarie. Adapted from https://commons.wikimedia.org/wiki/Category:Allen–Lambe_House;/. Accessed on 10 January 2023 (**b**) Abstaction in the window ornmetation (**c**) Horizontal detailing to the landscape and garden walls.

A different approach is found in the case study as seen in Figure 5. This comprises- (a) the winter view shows multiple analogies of form and the prairie as a natural system, i.e., roof, masonry, and stone courses. (b) The window glazing shows an ornamental abstraction of natural elements tempered by machine manufacture. (c) Landscape elements are ordered in a similar way to the building, with the natural systems as a backdrop. Both are examples of bio-utilization in the Prairie system and the case study adds additional biomimetic dimensions to the design, that is, the analogies of nature existing in the shaping of all of its elements.

**Figure 6:**
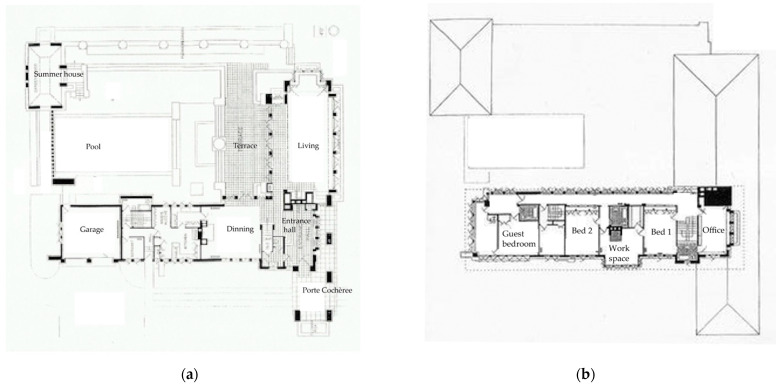
Allen–Lambe case. (**a**) Ground floor plan linear form: https://www.pinterest.com.au/pin/646336984041979486/ accessed on 10 January 2023; (**b**) Second floor: https://www.pinterest.com.au/pin/646336984041979343/. Accessed on 10 January 2023.

In complex design thinking, one of the challenges is how the whole is reflected in the parts; the concept of the Prairie system is abstracted as horizontal lines and these are reinterpreted in the window ornamentation and the roof; the aim is to create holism in design by integrating the concept of nature into every part. Figure 6 shows this linear approach in the floor. planning. 

This is manifested in the detailed planning. (a) The ground-floor plan: the east–west linear element contains the entrance, dining area, kitchen, and garage, while the north–south area contains the living area.

The summer house is in the northwest corner. The spatial planform is independent of the roof form. (b) The second floor contains the office, bedroom, and service areas, drawn from the environmental services provided by natural systems from the south in the winter and the north in the summer.

#### 3.3.2. Massing and Typological Differences

**Figure 7:**
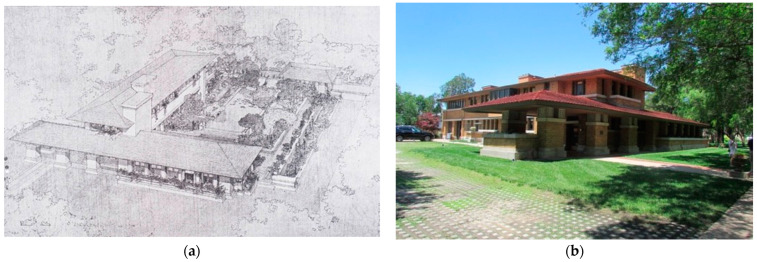
Allen–Lambe case: the massing of the case study. (**a**) Adapted from perspective view: https://www.pinterest.com.au/pin/646336984042470252/ accessed on 10 January 2023. (**b**) street view.

These dimensions come from Wright’s interpretations of the site. They appear to act as transformative ideas to create the character of the interior setting specific to the site and program, but one’s experience of nature is indeed very different for each building Figure 7. shows the massing of the case study.

This comprises the two-story linear mass which divides the site into a northern landscape courtyard and a southern courtyard. (a). The single-story mass to the east extends and separates a third eastern zone in the setback. (b) The street view is of the setback and the architectural intention is to incorporate natural systems as buffers between the buildings and the street. This manifests in the Allen–Lambe analysis of massing and typological comparisons (see Table 2). 

#### 3.3.3. Bio-Systems Analysis

**Figure 8:**
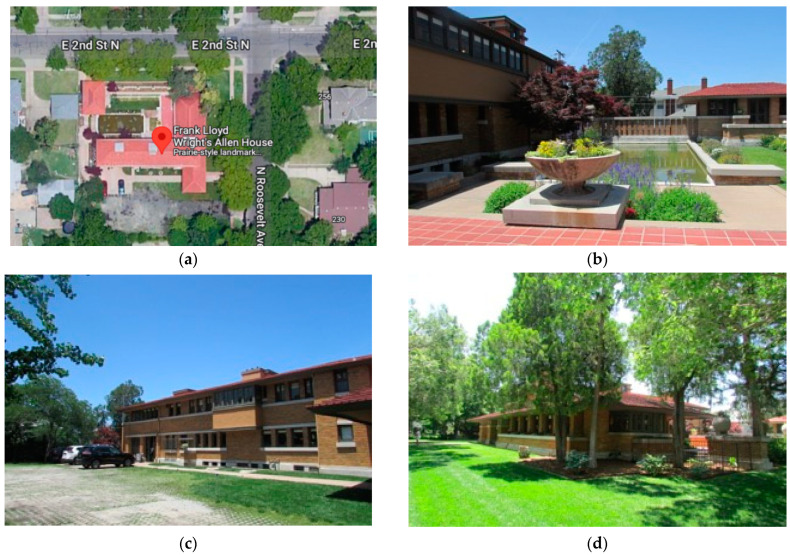
Allen Lambe case. (**a**) The site planning; adapted from: Google Maps Commons. https://www.google.fr/maps/dir/Frank+Lloyd+Wright’s+Allen+House,+255+N+Roosevelt+St,+Wichita,+KS+67208,+United+States//@37.6898506,-97.3274288,13z/data=!4m13!4m12!1m5!1m1!1s0x87bae31355ea1d9b:0xc467fefb15016d41!2m2!1d-97.2922664!2d37.6897874!1m0!2m3!6e0!7e2!8j1683878400!3e0?hl=en. Accessed on 10 January 2023. (**b**) northern couryard; (**c**) southern courtyard; (**d**) eastern setback from the street.

Figure 8 shows a more detailed systems analysis from the ‘bio’ perspective starting with the site, strategy and shell optimization for natural systems.

Site—Located in a warm-temperate continental climate, it has hot summers with humid conditions and cold winters; on suburban the site in Wichita, predominantly deciduous forested trees are found. The primary two-story form intersects with a single story.

This is divided into three zones, the northern quadrant is service-orientated, the easterly quadrants contain a street setback, and the southern quadrant is the ornamental courtyard. Each of these zones has very different bioclimatic ecological characteristics and, in this way, they allow the appropriation of the building from the street and to the broader ecosystem and vice versa. (See Appendix A).

The summer conditions are shown here: (a) Site plan indicates a northern landscaped courtyard and pool, a southern service courtyard with planning in the eastern setback. (b) The southern courtyard is a summer space due to solar access, forest trees at the boundary control airflow, the shell is shaded, and ventilation and evaporative cooling to the open space. (c) Southern courtyard without landscape and deciduous trees which lose their leaves allow solar access. Shell provides shading to windows for summer control and ventilation. (d) Easterly landscape zone a mixture of forest and grass to provide shade in summer. The bio-utilization benefits include cooling from transpiration from the vegetation.

Strategy—Sitting on a 1-acre block and with comparatively small programmatic requirements, as a small family with functionally modest artistic tastes, the site provided little constraints to the building form; however, the form is interdependent with the site zones for environmental services, ventilation, solar heating, and daylighting, following the master planning from the prairie housing model for suburban housing.

Shell—This plan and linear form are different from the cruciform prairie housing model; this increases environmental exposure, maximizing environmental services (see Appendix B). The shell is optimized biomimetically to work with these site zones; the form of the envelope is adapted very differently in each elevation: the south is thin and transparent, and the north is deep and thick, reflecting the heat gradient across the section. The roof is ordered accordingly to achieve the symbolic constraints and biomimetics through the abstraction of the prairie ecosystem and topography.

**Figure 9:**
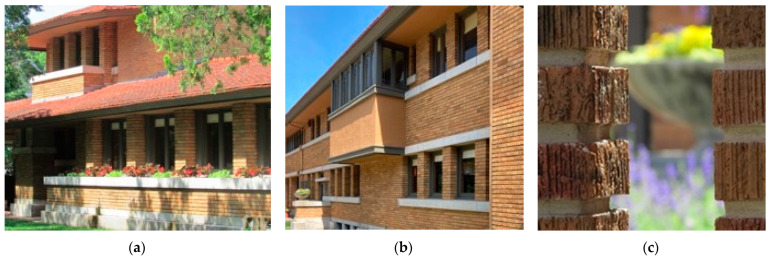
Allen Lambe case. Structure and skin systems. (**a**) Biomimetic integrating the natural analogue and technical innovation. Skin adaptations—(**b**) The use of materials is at different scales, including masonry functions such as the ecosystem responding to the diurnal and seasonal changes for mutual benefit. It should be noted that the ornamental detailing of brickwork, vertical surface raking, and (**c**).horizontal jointing intersect with sunlight to create shade and shadow effects on the façade and permeability to perimeter walls.

Figure 9 shows the biomimetic integration of the natural analogue, technical innovation and natural systems in the structure and skin.

Structure—The foundations and bearing structure are integral to the skin and reflect symbolic elements.

Skin—External surfaces are mostly masonry and stone and are substantive with high thermal mass. This is very important to insulate the building, particularly when the shell has a geometry that increases environmental exposure to factors such as daylighting and ventilation (see Appendix B climate and psychrometric analysis). (a) Additionally, they are ordered to achieve symbolic constraints. The articulation of the skin, as with the roof, is decorated as a mimetic prairie via abstraction.

**Figure 10:**
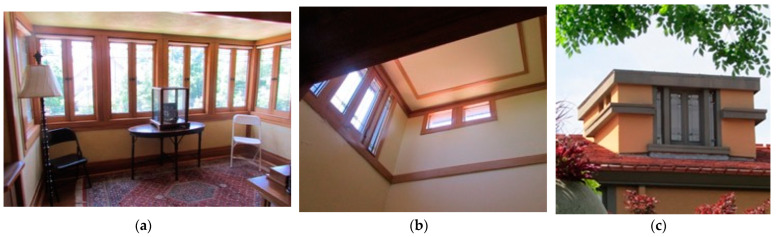
Allen Lambe case. Services and integration, roof monitors for access to light, and ventilation to the bathroom and bedroom study area.

Services—HVAC, electrical, mechanical, and hydronic systems are integrated within the envelope; however, the fireplace is articulated to achieve symbolic constraints. Vertical light and ventilation shafts are provided to the second-floor bedrooms, as shown in Figure 10. Early adoption of new technological innovations for home comforts is included, such as centralized vacuum systems, gas log fires and alarm systems [49].

**Figure 11:**
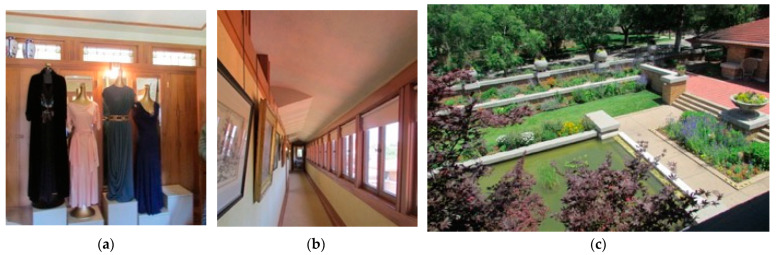
Allen Lambe case. Space planning and biophilic connections.

**Space plan**—Figure 11 shows an example of the liner spatial organization, with indeterminant bioclimatic circulation connected to the site zones for light, air, and heat, and in the biophilic sense, the experience of the view of the natural environment. The most used spaces are adjacent to each other and offer easy accessibility.

This multi-layered approach is seen in the bio-utilization of environmental services and potential biophilic connections that show the degree to which nature is part of the building.

**Figure 12:**
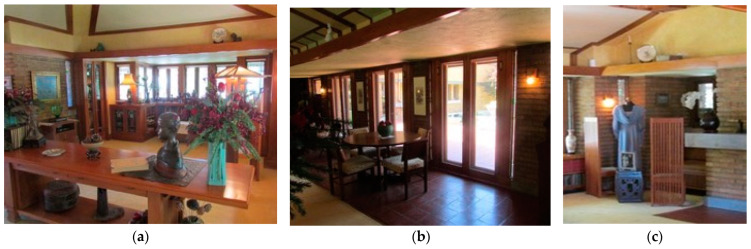
Allen Lambe case. Space plan, scenery, set, and stuff in the living area.

As seen in Figure 12 the creation of ‘spaces within spaces’ is a phenomenon that adds to the nature of the living room setting, including: (a) the ceiling is carved out of the roof space, and a window bay appears to create a secondary space for reading with a lower ceiling height; (b) the breezeway through double doors bring cross ventilation from the courtyard; and (c) the snug, carved from the hearth.

**The scenery**—The layout of internal partitions is minimized, walls and ceiling reflect the external form, and the hearth and snug are exaggerated in size and were designed anthropomorphically, i.e., it includes anthropomorphic integration: the snug is a one- or two-person space.

**Set**—The furniture and equipment are bioinspired and support the symbolism. The furniture is integrated into the scenery and space planning.

**Stuff**—The occupants’ belongings are artefacts of artistic beauty.

An overall observation from this analysis is that Wright’s work is an example of bioinspired biomimicry and that the prairie system is a type that allows the design to be true to its type but adaptable to the site and program, and further, that ecosystem mimicry involves a perhaps fortuitous level of eco-integration. A summary of the comparison btewwn systems is seen in Table 2.

The typologies show differences in site and building planning and the potential environmental response. Wright’s reaction to vernacular planning was to increase environmental exposure in the building massing to better bring together the building, site, and occupant.

The Allen–Lambe house was built in 1916 [50]. Over a hundred years have passed, during which time the ecosystems surrounding it have been allowed to evolve. This enables us to see how this process enriches the design. Furthermore, comparing it with the site master planning based on Wright’s model for suburban living and that of the site planning of the case study, a new understanding of the ecological potential of Wright’s work is gained.

#### 3.3.4. Aligning Architectural Intent with Ecological Purpose

**Figure 13:**
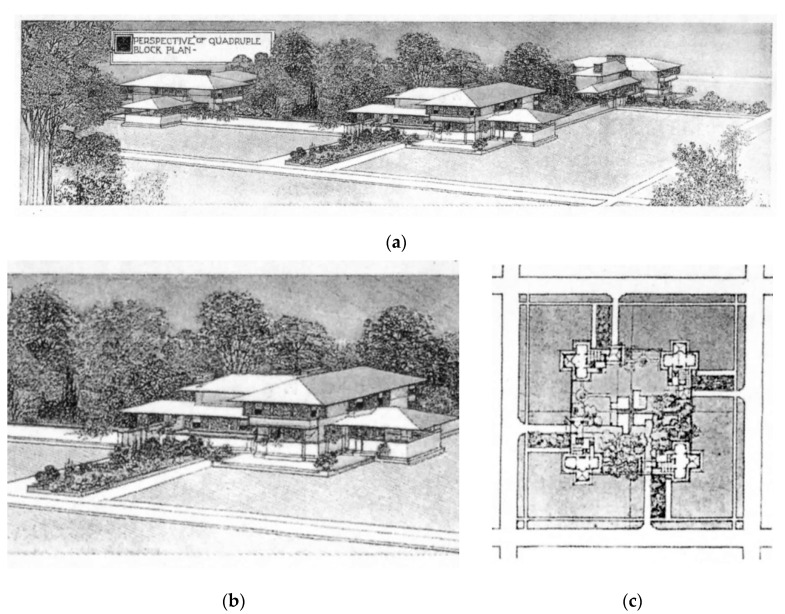
Prairie system intent. (**a**) The perspective of the block and the massing; (**b**) site master planning intent for a four-block cluster of Wright’s prairie ‘model’ for suburban living; (**c**) the potential ecological integration with a natural backdrop. Wright, Frank Lloyd, “*A Home in a Prairie Town*”, http://www.steinerag.com/flw/Periodicals/LHJ.htm. Accessed on 10 January 2023.

Wright’s architectural intent is found in the additional information on his model. He criticized urban housing for placing too much of the program in the house, compressing the site and distorting the planning. He advocated for the prairie model as a clustered arrangement of four houses, forming a community with a larger landscaped area, providing prospects from the community but also privacy through natural features and landscaping (Figure 13).

The constituents of the model are observed as follows:

1. Privacy from the public domain is achieved through distance from the boundaries. Service access is provided for vehicular and pedestrian paths with a garage at the center of the block. Privacy is achieved by increasing the density of the informal ecological spaces in the centre (a).
2. The grid at the urban scale and at the block scale are synchronized consistent with Wright’s Unit system and applied to the ornamental elements, access to roads and paths. A landscape zone dives pedestrian and vehicular assets; these divide the block into four quadrants, with houses at each corner oriented in two directions. Each house is standardised as a system but each has a different prospect. (b).
3. The peripheral vegetation at the edge of the block provides screening and blocks the line of sight from the public domain.
4. The clustering of the buildings for an ecological opportunity utilizing = this external landscape and relatively small building footprint should be noted. Wright portrays the ecosystem function as an informal landscape that allows nature to take its own path and aligns this with architectural intent. (c) [38].

One of his main propositions is integrating the building and site in both a romantic and a practical sense, harmonizing with nature through bio-utilization for its intrinsic values. This is recognized by matching the building and site′s form to the prairie′s massing. Typology aims to frame nature where the building and site share the same programmatic and functional requirements for modern living through informal ecological spaces and formal landscape spaces 

#### 3.3.5. Ecological Concepts

Further analysis of Wright′s thinking reinforces the form matching and program sharing between the site and building. At the time, mainstream architects believed that form followed function, and he challenged this notion by arguing that form and function can be combined as one; intrinsically, these are framed as the objectives of the design process. Analysis by Cruz argues that the highest achievement of this type of architecture is the continuity it brings to resolving the building and its environments [51].

**Figure 14:**
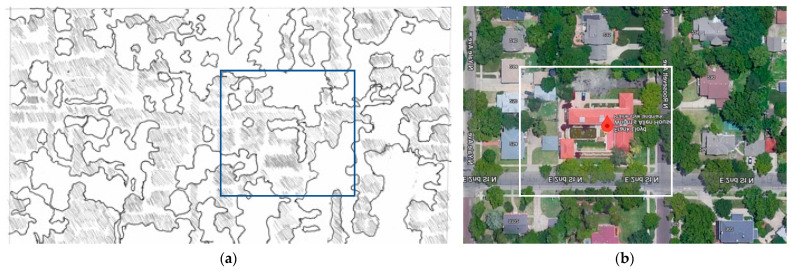
Allen-Lambe case. Ecological integration at varying scales. (**a**) Neighborhood scale; most biomass (whites zones) and least biomass, buildings, roads (shaded); (**b**) site scale. Google Maps Commons. https://www.google.fr/maps/dir/Frank+Lloyd+Wright’s+Allen+House,+255+N+Roosevelt+St,+Wichita,+KS+67208,+United+States//@37.6898506,-97.3274288,13z/data=!4m13!4m12!1m5!1m1!1s0x87bae31355ea1d9b:0xc467fefb15016d41!2m2!1d-97.2922664!2d37.6897874!1m0!2m3!6e0!7e2!8j1683878400!3e0?hl=en. Accessed on 10 January 2023.

Figure 14 shows the consequences of mutual benefit observed here inferring that the site provides spatial context for natural systems to mature, and the buildings then benefit from the services provided. Without the naturalism of the site, there is no function and there is no form. Hence, form and function are the ends of the design process, the making of the whole, and the other dimensions of materials and construction provide the means.

In Wright’s ideas for integrating architecture into nature, there is a preference for bio-utilization, mainly for the intrinsic quality of the materials, although this is tempered by the opportunities that the advances in technology offer. The construction is assembled and shaped by the site. This is made possible by the environmental building attributes influenced by the biophysical and bioclimatic constituents. The questions that arise from these attributes mimic and allow ecological integration. Do the organic and non-organic components, i.e., building form and site masterplan, allow ecological complexity, biomass growth and energy reduction, ecosystem succession, and maturation and eco-cycle integration.

#### 3.3.6. Ecosystem Mimicry and Eco-Integration

**Figure 15:**
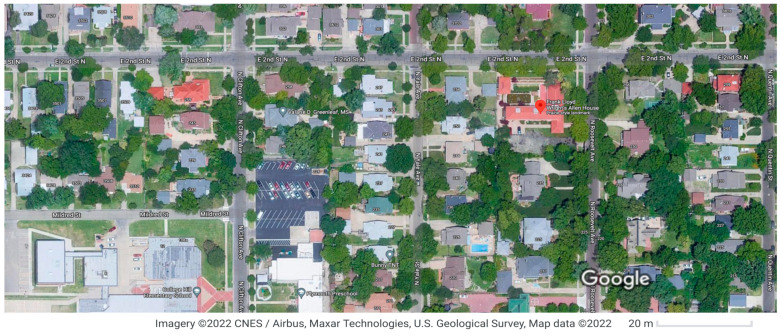
Allen-Lambe case. Ecological integration at the suburban scale: Google Maps Commons. https://www.google.fr/maps/dir/Frank+Lloyd+Wright’s+Allen+House,+255+N+Roosevelt+St,+Wichita,+KS+67208,+United+States//@37.6898506,-97.3274288,13z/data=!4m13!4m12!1m5!1m1!1s0x87bae31355ea1d9b:0xc467fefb15016d41!2m2!1d-97.2922664!2d37.6897874!1m0!2m3!6e0!7e2!8j1683878400!3e0?hl=en. Accessed on 10 January 2023.

Wright’s siting concepts, ecosystem mimicking and eco-integration, are used to analyse the case of the Allen–Lambie house, in terms of how it shapes a very different realization of the future of the building as shown in Figure 15.

In the Allen–Lambe house, he removes many of the constraints of the vernacular, allowing the architecture to establish several interdependencies between the internal and external spaces. Hence, how the site and associated ecosystem can be mutually beneficial. (**a**) Initial bio-utilization mapping shows boundaries to more intense ecosystems and physical urban infrastructure, such as landscaping, buildings, and foot roads. (**b**) Surrounding microclimates, the ecosystem enhances the form to create the northern and southern courtyards for the mutual benefit of the house.

The suburban morphology surrounding the site has evolving changes in land use and ecological impact, that is, changes to the biomass and energy balances of the potentially emerging forest ecosystem. Ecosystems are now replaced by car parks and mega forms such as institutional buildings. A way to reverse this trend is the conservation of ecosystems and utilization at the neighborhood and suburban levels.The implications of ecologic mimicry and integration for the building design are seen in further analysis; rather than run a simulation study, a psychrometric analysis of the Wichita warm temperate climate was carried out. The design strategies for passive active and microclimate control, determined climatically, are compared with those used in the Allen–Lambe house (see Appendix B).

#### 3.3.7. Form and Function

This plays out in shaping the site regarding its three site zones, as described above, in the Allen–Lambe house. This provides the infrastructure for both manmade and ecosystem elements. This is significant in the building planning that remains similar for each house; its different orientation offers each building privacy and a unique perspective of the site. The ecological providence of Wright’s work is now unfolding from understanding the importance of a forest ecosystem to a city. 

The case has evolved around these buildings made possible by the small building footprint-to-site ratio. At the building scale, increasing at neighborhood scale, a forest ecosystem is emerging and associated biomass impacts the building’s energy use. Krieger reports on the wide impimprovement, and environmental quality results from this type of ecosystem, including water quality, soil stabilization, air quality, climate regulation, carbon sequestration, and biodiversity [52]. 

**Figure 16:**
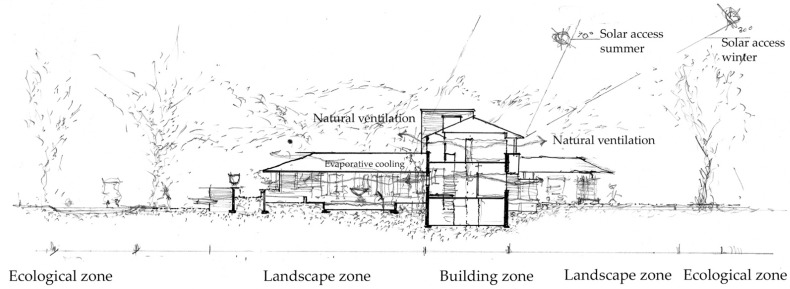
Allen- Lambe case. Site section north and south of the case study house. Biomimetic form and function like the adjacent biological and ecological systems. Shell and skin are adapted to natural systems of sun and winds such as shading to equator-facing surfaces, whole house ventilation and broader opportunities for ecological integration.

Rosland argues that, in fighting climate change, this is one of the major nature-based solutions available [53]. However, the linkage from the building scale to the neighbourhood and city scale is yet to be fully explored (see Figure 16).

#### 3.3.8. Material, Construction, and Process

Combining the site strategy and the materials, the construction and the interaction process have some environmental consequences. The mutually beneficial environmental effects of Wright’s work have been noted from a technical perspective through a simulation study by Geva. This study is one of only a few such explorations and comes from Wright’s early period in the cold climate of Chicago, It drew several conclusions:

1. The designs showed sensitivity to the thermal properties of the materials;
2. He experimented with thermal mass, such as masonry and concrete using their climate control mechanisms;
3. The horizontal cantilevered forms created effective shading systems;
4. Passive heating and cooling systems with effective heating systems removed many of the constraints found in the vernacular architecture [54].

These ideas, pre-1901, arguably set the foundation for Wright’s broader mimetic ideas of abstracting the form of the Prairie to his architecture involving the use of local materials such as timber and brick, which create the constituents of the typology. These are, in fact, metered by integrating the architecture into the site and its associated ecosystem and programmatic requests from the client; for example, the garage and outbuildings no longer occupy the central quadrant but are integrated into the form, and the master bedroom is increased in size and projects from the façade. This carving of the program into form is constant with the flexibility of the architectural system. The spatial integration can be seen in the north–south sections; the overhanging eaves are deleted in the requested garden. There are numerous adaptations in the orthodoxy of the typology, including in the snug, which creates a space within a space to provide comfort in the depth of winter, and the summer house in the northwest corner.

Of significance is the microclimate control created by the ecosystem integration and specific regulation to allow solar access to the private living space in the south-facing façade. The new microclimate of the walled sequestering garden creates a site-specific ecological opportunity with its terracing form and juxtaposition to street planting. Its pool creates an evaporative environment for the hot summer conditions; the south space is opened to provide solar access and ventilation to the southern façade, whilst still relating to the ecosystem maturing around it. The strategies are synchronous with modern guidelines for bioclimatic buildings for this climate.

## 4. Reflection

Wright’s broader bioinspired ideas about taking a part of nature such as the prairie landscape and using it as the basis for an architectural system uses biomimetic thinking in both the architectural problem and ecological solution spaces. This deeply imaginative process integrates related ‘bio’-perspectives such as bio-utilization, biophilia, bio-utilization, and the bioclimatic process. However, biophilic perspectives require further evidence to support the hypothesis. Karakas, and Yildiz’s research places this as the extension of behavioral research in architecture using neuroscience to understand this phenomenon [55].

The concept of form and function explains some of the interdependence between the building and site in the systems analysis methodology used in this study.

This is attributed to the prairie house site master planning, which was envisaged as a suburban housing model using a standardized building typology, with cluster buildings but orientated to different aspects and landscape settings, giving each building a unique view of the site whilst also securing privacy. In the Allen–Lambe case, this approach is taken further, where the standard building typology is revised and shaped by specific client programmatic requirements and biomorphically to the three site zones.

Furthermore, looking at Wright’s work from an ecosystems approach, it can be found that whilst the starting point of the design was the prairie landscape, a new forest ecosystem system appears to have evolved in and around the site. This is increasing potential biomass, stabilizing energy flows and improving the quality of the services of the site and the neighborhood.

The ecological providence that Wright envisages from the small foot building and large site area system is perhaps a legacy that can address the impact of climate change in this way. As seen in the site analysis, the urban morphology is increasingly eroding into this new ecosystem, rapidly changing the organic to the inorganic ratio. The question remains: how can this morphology be changed?

Wright’s conversation with society is a manifesto for urban planning based on the proposition of integrating urban planning into nature. This hypothesis being discredited at the time as utopian and impractical paved the way for 40 years of urban and suburban densification and what is arguably an urban dystopia [56].

More recently, this proposition has been reinvestigated. He believes that new modes of transport and communication made the current high-density cities less attractive and that life could be reimagined in the suburban, more rural environment [57]. As seen in this case study, the question arises: can a utopian and discredited model of suburban living, bioinspired and mimetically designed, have the potential to make a difference in the action against climate change and the erosion of the quality of life that will ensue?

Recent research has some positive support for this approach. The utilization of land—, i.e., the abiotic and the biotic uses in our cities, should be retrofitted around a bioinspired architectural systems-based manifesto. Central to this is the use of biomimetics, inter alia, related biofields: to inform the design of the parts of the whole and through natural systems, the whole can inform the parts. Research is advanced in this area; for example, work on biomimetic building skin systems demonstrates several methodologies which could be extended to a natural systems integration [58], and ecosystem conservation can be achieved by natural systems through a place-centered approach [59].

Whilst these systems use sustainability as a goal, this could be grounded in the demands, mitigating the sources of local environmental pressures and mitigating carbon pollution, addressed at the city scale via site-based vegetation. Biomass is represented by vegetation land cover across the city. Wei reports on the modelling of the impact of vegetation on biogenic CO_2_ flux in New York which has the largest anthropogenic CO_2_ emissions of any city in the US. This city appears to contain little vegetation. However, mapping what is still available, the ‘biogenic CO_2_ uptake still offsets up to 40% of the city’s CO_2_ enhancements from anthropogenic emissions on summer afternoons’ [60]. Every bit of ‘green’ matters.

## 5. Conclusions

Current perspectives of nature are developing in ecological design sourced from the roots of ecology, leading to organic architecture at the turn of the century, aimed at integrating architecture into nature and the mutual benefits that come with this. Recently, the systems approach to ecological design, aimed at emulating nature, is leading to a synthetic perspective of nature, where manmade environments are preferred. New, pragmatic types of buildings that are highly mechanically serviced and energy-dependent are developing, displacing environmental design typologies and increasing the pressure on the environment. Such is the concern of the schism between social and ecological paradigms that a new perspective that darkens our view of nature is emerging due to failures to address global-scale impacts.

New perspectives such as bioinspired architecture based on biomimicry promise a new direction. The study compares bioinspired architecture with its early predecessor, organic architecture; whilst sharing inspiration from nature, their objectives are different, the former integrating nature into parts of architecture, and the latter aiming to integrate architecture into nature. Similarities are found in sources of ideation in nature using biomimetics and related bio-concepts such as bio-utilization, biophilia, and the bioclimatic. Furthermore, all of these use design processes of abstraction and interpretation, share problem–solution spaces in nature and, share a systems approach. The difference is largely in the realization of the whole, which demands an adaptability typology responding to the program′s constraints, function, site integration, and environment.

Designing methodologies for mimicry involving working in different architectural, technological, biological, and ecological knowledge domains are challenging. Research exploring methodologies that are aimed at aligning architectural intent with biological and, more broadly, ecological purposes are needed.

The bio-architectural systems approach explored here is a possibility for addressing these challenges. Founded in systems thinking associated with the case study building, it is possible to explore the interdependence of the current biomimetic and ecological parameters and the architectural strategies used.

The case study demonstrates the significance of the site and its broader ecological milieu as a source of ideation, for abstraction and interpretation leading to realization. Whilst the vernacular architecture, in comparison, appears pragmatically grounded in function, the case study building extends the complexity to a bespoke, eco-mimetic solution, aligning function, form, process, site, and ecosystem. The challenge for future research is to embrace Wright‘s vision for a contemporary model of suburban living based on bioinspired ecological design. This appears a feasible goal but requires a new social mindset to achieve.

## Figures and Tables

**Table 1:** Ecosystem mimicking and eco-integration for buildings and sites.

**Ends**	**Strategies**
Form	The building and site look like an ecosystem for example the amount of biomass and energy use.
Function	The building can function in the same way that an ecosystem would and forms part of a complex system by utilizing the relationships between processes; it is able to participate in the hydrological, carbon, and nitrogen cycles in a similar way to an ecosystem, for example, the extent that to which the building /site/ecosystems relationships are mutually beneficial.
**Means**	**Strategies**
Material	The building and site are made from the same kind of materials that ecosystem is made of; it uses naturally occurring common compounds, and water as the primary chemical medium, for example, the use of local materials.
Construction	The building and site are assembled in the same way as an ecosystem; principles of succession and increasing complexity over time are used, for example, the changes in species inhabiting the ecosystem.
Process	The building and site work in the same way as an ecosystem; they capture and converts energy from the sun, and stores water, for example, for the use of environmental services offered by the ecosystem.

**Table 2:** Comparison of the Allen–Lambe case, vernacular system, and Prairie system cases ^1^.

Ornamental Applications	Site and Building Planning and Massing	Program and Spatial Organization	Environmental Response
A vernacular kit housing case			
Figurative and use of decorative elements of the interior spaces. Functional use of materials.	Square compact three-story centrally placed on the site with porches, axil entrance to/from the street, and symmetrical planning.	Large family, four square rooms per floor, central hall, and stairs. Access to outdoors from the front and rear entrances	The compact form reduces environmental exposure and lightweight timber materials increase thermal response. Environmental control systems located on the exterior, i.e., chimneys and windows, are positioned by planning and façade symmetries.
Prairie system			
Abstract parts to whole and whole to parts from nature. This is applied for the interior and exterior forms	Cruciform plan, two-story, asymmetrically internal planning placed on the site to provide the natural backdrop and prospect to a landscaped space.	Large family accommodation, with an internal two-story galley, open planning on the ground floor more cellular planning on the second floor.	Less compact form increases environmental exposure. Heavyweight walls of masonry construction timber floors reduce thermal response. Central interval fireplaces and windows are positioned to allow light and ventilation.
Allen–Lambe house case			
Abstract parts to whole and whole to parts. Japanese cultural traditions are also used.	L-shaped linear asymmetrical planning and massing. Two-story and single-story interconnecting volumes placed centrally create different zones. The natural backdrop expanded into the service space and landscaped garden	Small family program with an office on the first floor. Open planning on the ground floor, with access to outside from living spaces cellular first floor	Strategies as above, with the exception that the linear form reduces plan depth and increases environmental exposure as the surface area increase. These adaptions to the site indicate a precise coupling with the site zoning.

^1^ Information sources from analysis of Prairie style planning and 182–187 and vernacular housing, Storrer p 49.

**Figure A1:**
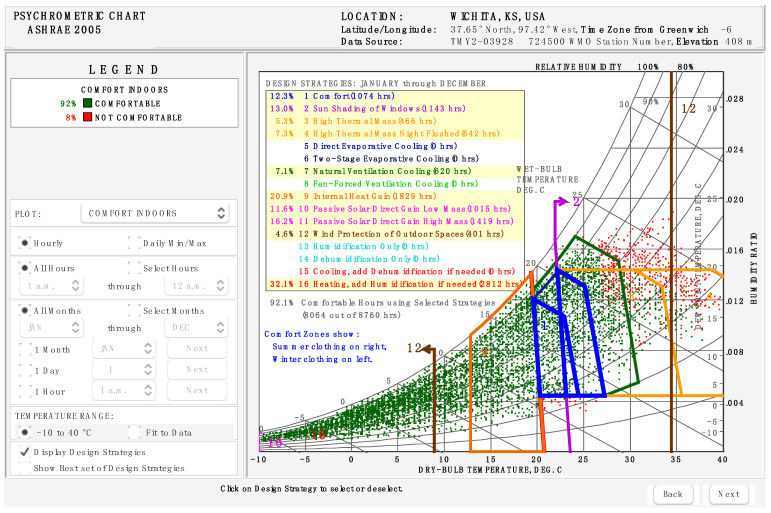
Modelling of the Wichita climate for comfort conditions using ASHRAE 2005 Psychrometric chart for Wichita. Adapted from Climate Consultant tool ©. https://energy-design-tools.sbse.org. Accessed on 13 April 2023.

## Data Availability

The author has attempted to trace the copyright holders of all material published in this article. If any copyrighted material has not been acknowledged, please let the author know so it can be rectified.

## Appendix A. Overview and Three-Dimensional Views of Case Study Spatial Elements

The links in this appendix were accessed on 10 January 2023.

Overview

https://www.visitwichita.com/blog/post/frank-lloyd-wright-house-wichita/

https://www.google.com/maps/place/Frank+Lloyd+Wright’s+Allen+House/@37.689787,-97.3011649,15z/data=!4m9!1m2!11m1!3e4!3m5!1s0x87bae31355ea1d9b:0xc467fefb15016d41!8m2!3d37.6897834!4d-97.2922665!15sCgEqWgMiASqSAQZtdXNldW3gAQA.

Frank Lloyd Wright’s Allen House,255 N Roosevelt St, Wichita, KS 67208, United States.

Street view winter

https://www.google.com/local/place/fid/0x87bae31355ea1d9b:0xc467fefb15016d41/photosphere?iu=https://streetviewpixels-pa.googleapis.com/v1/thumbnail?panoid%3DIQSPL1n38P5RKpbt3na2fA%26cb_client%3Dlu.gallery.gps%26w%3D160%26h%3D106%26yaw%3D295.43277%26pitch%3D0%26thumbfov%3D100&ik=CAISFklRU1BMMW4zOFA1UktwYnQzbmEyZkE%3D

Street view summer

https://www.google.com/local/place/fid/0x87bae31355ea1d9b:0xc467fefb15016d41/photosphere?iu=https://lh5.googleusercontent.com/p/AF1QipPoPAWSwvjC-h_hQM_I5HmxENd8txLWCo8xzIQn%3Dw160-h106-k-no-pi-10-ya240-ro0-fo100&ik=CAoSLEFGMVFpcFBvUEFXU3d2akMtaF9oUU1fSTVIbXhFTmQ4dHhMV0NvOHh6SVFu

Dining area view

https://www.google.com/maps/place/Frank+Lloyd+Wright’s+Allen+House/@37.6897851,-97.2923455,3a,75y,116.52h,88.06t/data=!3m8!1e1!3m6!1sAF1QipOVuCFc554usTBHpsNhUiWvvh9NDLmyIyNPb8oP!2e10!3e11!6shttps:%2F%2Flh5.googleusercontent.com%2Fp%2FAF1QipOVuCFc554usTBHpsNhUiWvvh9NDLmyIyNPb8oP%3Dw203-h100-k-no-pi0-ya120-ro0-fo100!7i7680!8i3840!4m11!1m2!11m1!3e4!3m7!1s0x87bae31355ea1d9b:0xc467fefb15016d41!8m2!3d37.6897834!4d-97.2922665!14m1!1BCgIgARICCAI!15sCgEqWgMiASqSAQZtdXNldW3gAQA

Garden pool view—north zone

https://www.google.com/maps/place/Frank+Lloyd+Wright’s+Allen+House/@37.6898242,-97.2923996,3a,86.4y,251.63h,100.87t/data=!3m8!1e1!3m6!1sAF1QipNxrqFJNBKP1sfXWs3gBxE_4upPh33Xy5LD4vHN!2e10!3e11!6shttps:%2F%2Flh5.googleusercontent.com%2Fp%2FAF1QipNxrqFJNBKP1sfXWs3gBxE_4upPh33Xy5LD4vHN%3Dw203-h100-k-no-pi-23.950338-ya237.89087-ro-3.1822462-fo100!7i7680!8i3840!4m11!1m2!11m1!3e4!3m7!1s0x87bae31355ea1d9b:0xc467fefb15016d41!8m2!3d37.6897834!4d-97.2922665!14m1!1BCgIgARICCAI!15sCgEqWgMiASqSAQZtdXNldW3gAQA

Street East zone

https://www.google.com/maps/place/Frank+Lloyd+Wright’s+Allen+House/@37.6897851,-97.2923455,3a,90y,42.44h,100.24t/data=!3m8!1e1!3m6!1sAF1QipMp5VNigomKatEM44IObfQ0w-sSxBKWH2gUXDjl!2e10!3e11!6shttps:%2F%2Flh5.googleusercontent.com%2Fp%2FAF1QipMp5VNigomKatEM44IObfQ0w-sSxBKWH2gUXDjl%3Dw203-h100-k-no-pi-10-ya40-ro0-fo100!7i7680!8i3840!4m11!1m2!11m1!3e4!3m7!1s0x87bae31355ea1d9b:0xc467fefb15016d41!8m2!3d37.6897834!4d-97.2922665!14m1!1BCgIgARICCAI!15sCgEqWgMiASqSAQZtdXNldW3gAQA

Living room—note hearth and snug

https://www.google.com/maps/place/Frank+Lloyd+Wright’s+Allen+House/@37.6897851,-97.2923455,3a,90y,42.44h,100.24t/data=!3m8!1e1!3m6!1sAF1QipMp5VNigomKatEM44IObfQ0w-sSxBKWH2gUXDjl!2e10!3e11!6shttps:%2F%2Flh5.googleusercontent.com%2Fp%2FAF1QipMp5VNigomKatEM44IObfQ0w-sSxBKWH2gUXDjl%3Dw203-h100-k-no-pi-10-ya40-ro0-fo100!7i7680!8i3840!4m11!1m2!11m1!3e4!3m7!1s0x87bae31355ea1d9b:0xc467fefb15016d41!8m2!3d37.6897834!4d-97.2922665!14m1!1BCgIgARICCAI!15sCgEqWgMiASqSAQZtdXNldW3gAQA

South elevation

https://www.google.com/maps/place/Frank+Lloyd+Wright’s+Allen+House/@37.689787,-97.2924102,3a,75y,90t/data=!3m8!1e2!3m6!1sAF1QipN4BmknO8H-chIq9p1bVV5JP5-WPGPT4runBsLU!2e10!3e12!6shttps:%2F%2Flh5.googleusercontent.com%2Fp%2FAF1QipN4BmknO8H-chIq9p1bVV5JP5-WPGPT4runBsLU%3Dw203-h114-k-no!7i2048!8i1152!4m11!1m2!11m1!3e4!3m7!1s0x87bae31355ea1d9b:0xc467fefb15016d41!8m2!3d37.6897834!4d-97.2922665!14m1!1BCgIgAQ!15sCgEqWgMiASqSAQZtdXNldW3gAQA

North Elevation

https://www.google.com/maps/place/Frank+Lloyd+Wright’s+Allen+House/@37.6897805,-97.2922684,3a,75y,90t/data=!3m8!1e2!3m6!1sAF1QipOiRgIZfbNsI1HYQhF-1T2BWl-m0PSSVyk1rYLn!2e10!3e12!6shttps:%2F%2Flh5.googleusercontent.com%2Fp%2FAF1QipOiRgIZfbNsI1HYQhF-1T2BWl-m0PSSVyk1rYLn%3Dw203-h152-k-no!7i4000!8i3000!4m11!1m2!11m1!3e4!3m7!1s0x87bae31355ea1d9b:0xc467fefb15016d41!8m2!3d37.6897834!4d-97.2922665!14m1!1BCgIgAQ!15sCgEqWgMiASqSAQZtdXNldW3gAQA

## Appendix B. Case Study Climatic and Psychrometric Data

Wichita Kansas, USA is located in a warm temperate climate with precipitation creating humid conditions for most of the year. Its continental location makes for cold winters and hot summers. With a Koppen classification, Cfa is shared by other countries such as China and Japan and Australia [61].

Figure A1 shows climate analysis using the psychrometric process for the thermal strategies that potentially can be used in the case study building using Climate Consultant [62].

Figure A1 shows the climate analysis using the psychrometric process for the thermal strategies that potentially can er used in the case study building. The role of the microclimate is important, for example, 8% of the year are days of discomfort (noted in red), This can be compensated for with strategies provided by the courtyard space to the northern summer. The water feature potentially provides an evaporative cooling environment. This courtyard is created with buildings, landscapes and natural elements which provide wind protection.

- For winter: thermal mass, passive solar heating, use of internal heat gains, and active heating system [63].
- For summer: natural ventilation. mechanical ventilation, active cooling evaporative system. The guidelines recommend further site planning strategies to mitigate the hot dry summer conditions, and microclimate control such as the use of deciduous trees and shaded courtyards, with water features.
- Additionally, whole-house ventilation encourages air and heat movement both vertically and horizontally through the structure. Whilst more can be discussed about the building science of this building perhaps most surprising is the ecological providence of the prairie houses.

Based on further reflection on the ecosystem implications of the site planning in Wright’s vision for the prairie houses and its realization in the Allen-Lambe house in Wichita requires discussion as seen in Table A1. This table shows strategies for using the environmental services of the site and climate match techniques in the building. This form-matching process is indicative of a possible forward-looking agenda for conserving the building in line with the enviornamtal intent of the architect and for conservation of the microclimate in which its is situated

**Table A1:** Design strategies for warm temperate continental climates compare to the techniques used in the Allen Lambire case study *.

Elements	Strategies	Techniques in Case Study	Notes
General	High thermal mass, earth sheltered	Masonry construction for walls, floors ground connected with timber floors	Basement under living areas
Roofs	Light color to reflect solar radiation	Terracotta roof tiles	The roof is recently renovated
	Controlled low level ventilation	N/A	
	Bulk insulation or RFL to reduce upward heat flow	N/A	
	RFL under roof reduce downward heat transfer	N/a	
Walls	Windows shaded to exclude summer sun and accept	Roof creates overhanging eaves	
	Bulk insulation or RFL to reduce inward and outward heat flow	N/A	
	Moderate thermal mass both external and internal data	The thermal mass in external walls act as capacitive insulation as insulation effect	Thermal mass is used extensively in the chimneys and ventilators
Shape	Long narrow for warm temperate climates	Long narrow with integrated circulation space internally, second floor is single side circulation	All habitable rooms access external spaces.
Orientation	East—west axis windows facing equator	The main two story building is aligned to East–west, the single story is aligned north south.	Eastern windows have overhanging eaves
Ventilation	Close attention to be paid to control exfiltration an infiltration of air	Cross ventilation to all rooms and addition ventilation thought light air wells to bathrooms	Airconditioning has been retrofitted
Daylighting	Narrow plan makes good daylighting easy	Narrow plan form is used for both	South diffused light is used in bathroom and second floor gallery.
	Possible glare from winter solar access	Overhanging eaves used	

* Adapted from [64].

## References

[1] Wadley D. (2019). The City of Grace: An Urban Manifesto.

[2] Papanek V. (1995). The Green Imperative: Ecology and Ethics in Design and Architecture.

[3] Hay P.R. (2002). Main Currents in Western Environmental Thought.

[4] Kallipoliti L. (2018). History of Ecological Design.

[5] Uchiyama Y., Blanco E., Kohsaka R. (2020). Application of biomimetics to architectural and urban design: A review across scales. Sustainability.

[6] Pedersen Zari M. Biomimetic approaches to architectural design for increased sustainability. Proceedings of the SB07 NZ Sustainable Building Conference.

[7] Ripley R.L., Bhushan B. (2016). Bioarchitecture: Bioinspired art and architecture—A perspective. Philos. Trans. R. Soc. A Math. Phys. Eng. Sci..

[8] Whitesides G.M. (2015). Bioinspiration: Something for everyone. Interface Focus.

[9] Hallmark Research Initiatives, Melbourne. https://research.unimelb.edu.au/research-at-melbourne/multidisciplinary-research/hallmark-research-initiatives/bioinspiration.

[10] Das S., Ahn B.K., Martinez-Rodriguez N.R. (2018). Biomimicry and Bioinspiration as Tools for the Design of Innovative Materials and Systems. Appl. Bionics Biomech..

[11] Imani N., Vale B. (2022). Developing a Method to Connect Thermal Physiology in Animals and Plants to the Design of Energy Efficient Buildings. Biomimetics.

[12] Imani N., Vale B. (2022). Heating with Wolves, Cooling with Cacti: Thermo-Bio-Architectural Framework (ThBA).

[13] Biomimicry Institute What Isn’t Biomimicry?. https://biomimicry.org/what-is-biomimicry/.

[14] Wilson E.O. (1986). Biophilia.

[15] Hoyos C.M., Fiorentino C. (2017). Bio-utilization. Int. J. Des. Objects.

[16] Ryan C.O., Browning W.D., Clancy J.O., Andrews S.L., Kallianpurkar N.B. (2014). Biophilic design patterns: Emerging nature-based parameters for health and well-being in the built environment. ArchNet-IJAR: Int. J. Archit. Res..

[17] William B., Catherine R., Joseph C. (2014). Patterns of Biophilic Design.

[18] Olgyay V. (2015). Design with climate. Design with Climate.

[19] Pedersen Zari M. (2018). Regenerative Urban Design and Ecosystem Biomimicry.

[20] Badarnah L., Kadri U. (2015). A methodology for the generation of biomimetic design concepts. Archit. Sci. Rev..

[21] Farel R., Yannou B. Bio-inspired ideation: Lessons from teaching design to engineering students. Proceedings of the 19th International Conference on Engineering Design (ICED13).

[22] Thomas R. (1996). Environmental Design: An Introduction for Architects and Engineers.

[23] Yeang K. (2015). From Bioclimatic design to Ecodesign. Design with Climate.

[24] Gosling J., Sassi P., Naim M., Lark R. (2013). Adaptable buildings: A systems approach. Sustain. Cities Soc..

[25] Duffy F., Worthington J. (1972). Designing for Changing Needs. Built Environ..

[26] Leaman A., Bill B. (2004). Flexibility and adaptability. Designing Better Building.

[27] Hassan E. (2017). A review of flexibility and adaptability in housing design. Int. J. Contemp. Archit..

[28] Brand S. (1995). How Buildings Learn: What Happens After They′re Built.

[29] Habraken N.J. (1972). Supports: An Alternative to Mass Housing.

[30] Li G., Zhou J., Wang L. (2019). A Hierarchical Approach for the Sustainability of Residential Building Regeneration. Open J. Soc. Sci..

[31] Margalef R. (1963). On certain unifying principles in ecology. Am. Nat..

[32] Cuce E., Nachan Z., Cuce P.M., Sher F., Neighbour G.B. (2019). Strategies for ideal indoor environments towards low/zero carbon buildings through a biomimetic approach. Int. J. Ambient Energy.

[33] Anon, Praire School, World Heritage Encylopedia. http://community.worldheritage.org/articles/Prairie_style.

[34] Learned R., Ellington H. Frank Lloyd Wright’s, Allen House in Wichita. https://flwrightwichita.org.

[35] Walker R.A. (1956). Frank Lloyd Wright: His Contribution to Our American Culture. Land Econ..

[36] Wright F.L., Bruce B.F. (2008). The Essential Frank Lloyd Wright.

[37] Laseau P., James T. (1991). Frank Lloyd Wright: Between Principles and Form.

[38] Thompson I. (2002). Frank Lloyd Wright: A Visual Encyclopedia.

[39] Secrest M. (1998). Frank Lloyd Wright: A Biography.

[40] Wright F.L. (1975). The Cause of Architecture.

[41] Wright F.L. (1901). A Home in a Prairie Town. Ladies Home Journal.

[42] Hanks D.A., Frank L.W., Renwick G. (1999). The Decorative Designs of Frank Lloyd Wright.

[43] Goldscmidt G. (1988). Interpretation: Its role in architectural designing. Des. Stud..

[44] Eugene S. Biomimicry, When Nature and Technology Work Together. https://surbanajurong.com/perspective/biomimicry-when-nature-and-tech-work-together/.

[45] Etlin R.A. (1994). Frank Lloyd Wright, and Le Corbusier: The Romantic Legacy.

[46] Koning H., Julie E. (1981). The language of the prairie: Frank Lloyd Wright’s prairie houses. Environ. Plan. B Plan. Des..

[47] Storrer W.A. (2015). Frank Lloyd Wright: Creating American Architecture.

[48] Cooke A., Friedman A. (2001). Ahead of their time: The Sears catalogue prefabricated houses. J. Des. Hist..

[49] Anon (2006). What’s Wright About Wichita.

[50] Storrer W.A. (2017). The Architecture of Frank Lloyd Wright.

[51] Cruz C.A. (2012). Wright’s organic architecture: From ‘Form follows function’ to ‘Form and function are one’. Cloud–Cuckoo-Land. J..

[52] Krieger D.J. (2001). Economic Value of Forest Ecosystem Services: A Review.

[53] Roeland S., Moretti M., Amorim J.H., Branquinho C., Fares S., Morelli F., Niinemets Ü., Paoletti E., Pinho P., Sgrigna G. (2019). Towards an integrative approach to evaluate the environmental ecosystem services provided by urban forest. J. For. Res..

[54] Geva A. (1999). Frank Lloyd Wright′s Oak Park Architecture: A Computerized Energy Simulation Study. Technology in Transition: Mastering the Impacts.

[55] Karakas T., Yildiz D. (2020). Exploring the influence of the built environment on human experience through a neuroscience approach: A systematic review. Front. Archit. Res..

[56] Grabow S. (1997). Frank Lloyd Wright and the American City: The Broadacres Debate. J. Am. Inst. Plan..

[57] Wright F.L. (2020). Broadacre City: A New Community Plan. The City Reader.

[58] Al-Obaidi K.M., Ismail M.A., Hussein H., Rahman A.M.A. (2017). Biomimetic building skins: An adaptive approach. Renew. Sustain. Energy Rev..

[59] Kibler K., Cook G., Chambers L., Donnelly M., Hawthorne T., Rivera F., Walters L. (2018). Integrating sense of place into ecosystem restoration: A novel approach to achieve synergistic social-ecological impact. Ecol. Soc..

[60] Wei D., Reinmann A., Schiferl L.D., Commane R. (2022). High resolution modeling of vegetation reveals large summertime biogenic CO2 fluxes in New York City. Environ. Res. Lett..

[61] Koppen Climate Classification for Witchia. https://www.weatherbase.com/weather/weather-summary.php3?s=5427&cityname=Wichita,+Kansas,+United+States+of+America.

[62] Milne M., Liggett R., Benson A., Bhattacharya Y. Climate Consultant 4.0 develops design guidelines for each unique climate. Proceedings of the 38th ASES National Solar Conference.

[63] Zöld A., Szokolay S.V. (1997). Thermal Insulation.

[64] Greenland J. (1998). Foundations of Architectural Science: Heat, Light, Sound.

